# Supplementary material for: Characterization of a Phage-Encoded Depolymerase Against Klebsiella pneumoniae K30 Capsular Type and Its Therapeutic Application in a Murine Model of Aspiration Pneumonia
Source: Viruses. 2025 Oct 30;17(11):1446. doi: 10.3390/v17111446 (PMC12656996; doi:10.3390/v17111446)
Supplement: Supplementary file 1 [file viruses-17-01446-s001.zip › Supplementary Material.pdf]

Supplementary Material

Figure S1 Comparative analysis of ORF5 from phage phiTH1 and its homolog ORF37 from phage K5-2. (**A**) Sequence alignment and secondary structure comparison of ORF5 and ORF37 using ESPript 3.0. (**B**) Three-dimensional structure comparison between ORF5 (cyan) and ORF37 (pink) using predicted models from AlphaFold3 and PyMOL 2.6.1 for visualization. Pectate lyase of ORF5 was showed as green and pectate lyase of ORF37 was showed as red.

Figure S2 Establishment of the infection model and histopathological assessment. (**A**) Determination of the optimal infectious dose and therapeutic efficacy of Dop5. Survival curves of mice following aerosol challenge with varying doses (1×10^8, 2.5×10^8, and 5×10^8 CFU) of *K. pneumoniae* TH1. The dose of 2.5×10^8 CFU was selected as it represented the minimal dose that consistently resulted in 100% mortality within 7 days. The 50 μg dose (2 mg/kg based on a 25 g mouse) was selected as the experimental regimen for subsequent studies, as it significantly improved survival rates in the lethal pneumonia model. (**B**) Anatomy and H&E staining (200× and 400× magnifications) of organs from mice in different groups.

Table S1 Bacterial strains, plasmids and primers used in this study.
