# Supplementary material for: Characterization of a Phage-Encoded Depolymerase Against Klebsiella pneumoniae K30 Capsular Type and Its Therapeutic Application in a Murine Model of Aspiration Pneumonia
Source: Viruses. 2025 Oct 30;17(11):1446. doi: 10.3390/v17111446 (PMC12656996; doi:10.3390/v17111446)
Supplement: Supplementary file 1 [file viruses-17-01446-s001.zip › Table S1.pdf]

Table S1 Bacterial strains, plasmids and primers used in this study.

Strain/Plasmid/Primer	Description	Source/Purpose
K. pneumoniae		
TH1	Wild type strain of HiAlc Kpn isolated from NAFLD&ABS patient; K30-type *K. pneumoniae* strain	（1）
W14	Wild type strain of HiAlc Kpn isolated from NAFLD&ABS patient; K30-type *K. pneumoniae* strain	This study
A552	K30-type *K. pneumoniae* strain	This study
TH1_m1	*wcaJ* mutant in TH1	This study
TH1_m2	*wcaJ* mutant in TH1	This study
TH1_m3	*wcaJ* mutant in TH1	This study
TH1_m4	*wcaJ* mutant in TH1	This study
TH1_m5	*wcaJ* mutant in TH1	This study
Δ*wza*	TH1 deleted of *wza*	This study
TH1_m1/*wcaJ*	TH1_m1 complemented with the *wcaJ* gene	This study
TH1_m2/*wcaJ*	TH1_m2 complemented with the *wcaJ* gene	This study
TH1_m3/*wcaJ*	TH1_m3 complemented with the *wcaJ* gene	This study
TH1_m4/*wcaJ*	TH1_m4 complemented with the *wcaJ* gene	This study
TH1_m5/*wcaJ*	TH1_m5 complemented with the *wcaJ* gene	This study
A2	K1-type *K. pneumoniae* strain	This study
A25	K2-type *K. pneumoniae* strain	This study
A23	K5-type *K. pneumoniae* strain	This study
A66	K21-type *K. pneumoniae* strain	This study
A512	K24-type *K. pneumoniae* strain	This study
A1	K54-type *K. pneumoniae* strain	This study
A45	K64-type *K. pneumoniae* strain	This study
**Plasmid**		
pKO3-Km	Gene replacement plasmid derived from pKO3 with an insertion of Km resistance cassette into *AccI* site	（2）
pKO3-*wza*	pKO3 derivative, for *wza* deletion	This study
pET28a	Expression vector with an insertion of Km cassette	
**Primer**	**Sequence (5′→3′)**	**Function**
KO-*wza*-upF	GTACCCGGGGATCGCTTCCCACTCTGCCTTTGA TATCG	Construction of *wza* deletion
KO-*wza*-upR	TTCGGCATTAGCATTGGCAATTGGGACTGAAA CTACACGCTACA	
KO-*wza*-dnF	TGTAGCGTGTAGTTTCAGTCCCAATTGCCAATG CTAATGCCGAA	
KO-*wza*-dnR	ACTCTAGAGGATCGCAAACCTGGTGGTTACGC TGGGTG	
Dep(*orf5*)-F	GTGCCGCGCGGCAGCCATATGGATCAAGAAAT TAAAACAGTC	Purification of depolymerase ORF5

Dep(*orf5*)-R	CTCGAGTGCGGCCGCAAGCTTTTAGAACGTAG AAAGTGCAACCC

1. Yuan J, Chen C, Cui J, Lu J, Yan C, Wei X, Zhao X, Li N, Li S, Xue G, Cheng W, Li B, Li H, Lin W, Tian C, Zhao J, Han J, An D, Zhang Q, Wei H, Zheng M, Ma X, Li W, Chen X, Zhang Z, Zeng H, Ying S, Wu J, Yang R, Liu D. 2019. Fatty Liver Disease Caused by High-Alcohol-Producing Klebsiella pneumoniae. Cell Metab 30:675-688. 10.1016/j.cmet.2019.08.018.

2. Link AJ, Phillips D, Church GM. 1997. Methods for generating precise deletions and insertions in the genome of wild-type Escherichia coli: application to open reading frame characterization. J Bacteriol 179:6228-6237. 10.1128/jb.179.20.6228-6237.1997.
