# Supplementary figures and images for: Characterization of a Phage-Encoded Depolymerase Against Klebsiella pneumoniae K30 Capsular Type and Its Therapeutic Application in a Murine Model of Aspiration Pneumonia
Source: Viruses. 2025 Oct 30;17(11):1446. doi: 10.3390/v17111446 (PMC12656996; doi:10.3390/v17111446)

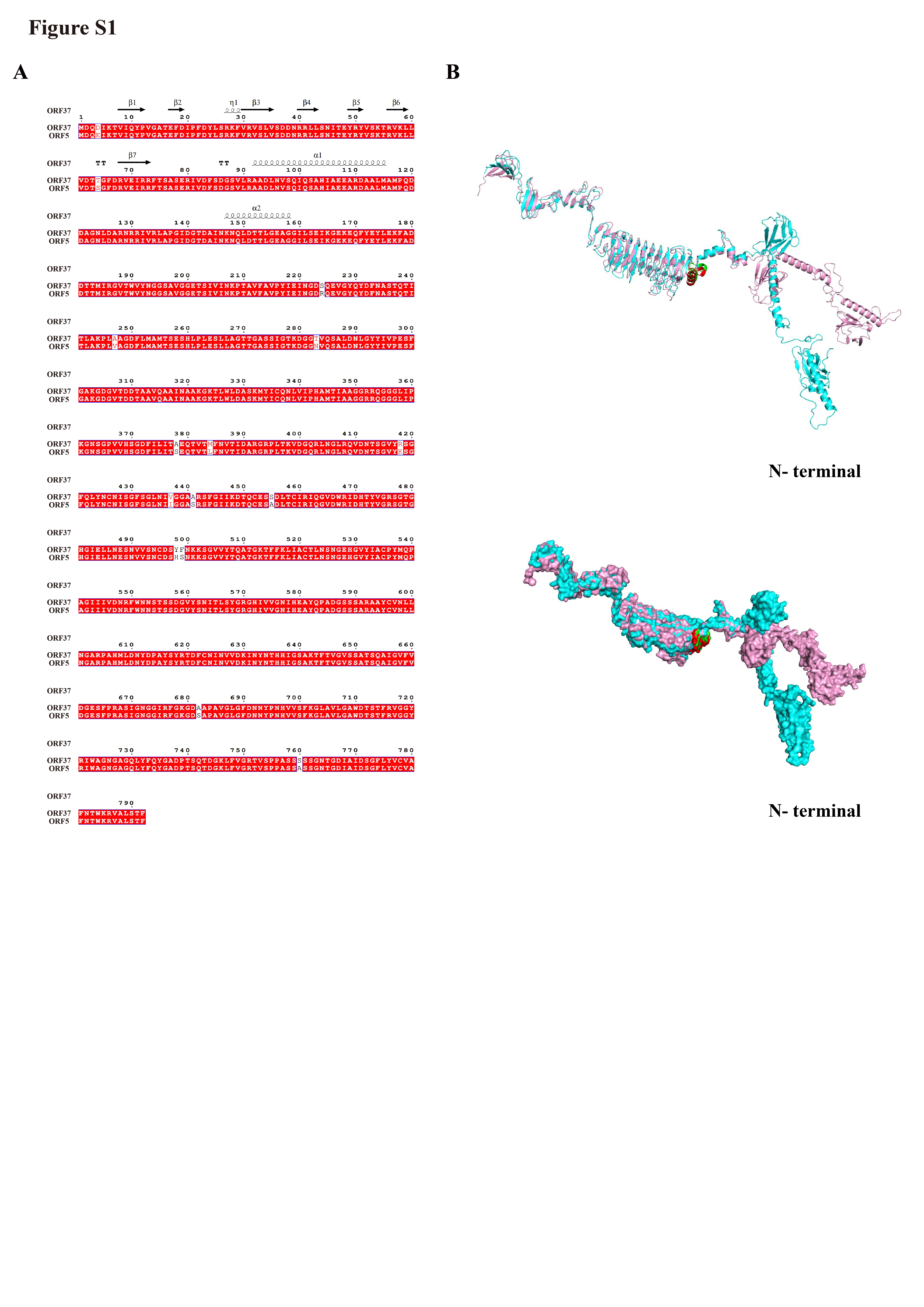

Supplement: Supplementary file 1 [file viruses-17-01446-s001.zip › Figure S1.jpg]

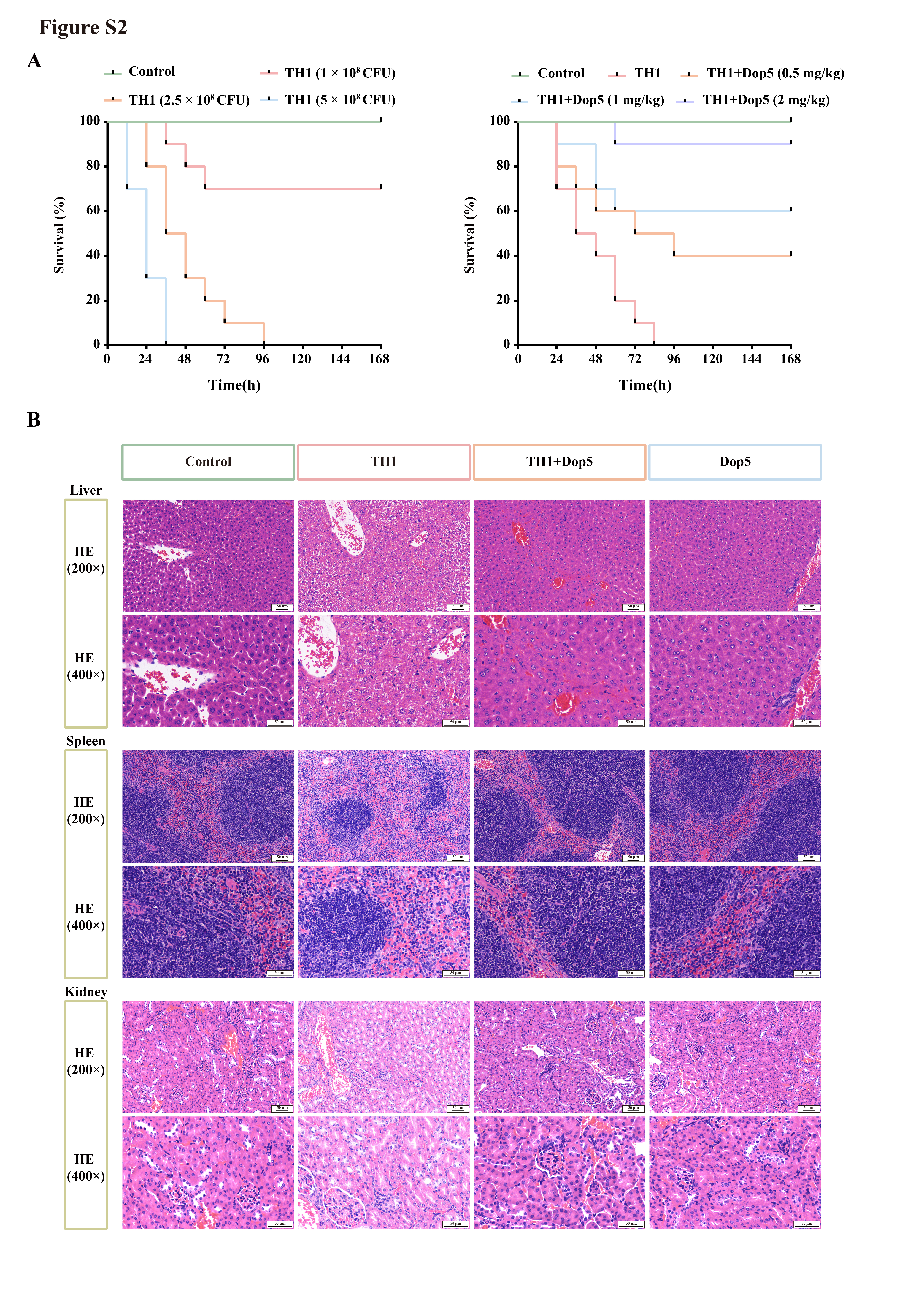

Supplement: Supplementary file 1 [file viruses-17-01446-s001.zip › Figure S2.jpg]
